# An annotated checklist of ladybeetle species (Coleoptera, Coccinellidae) of Portugal, including the Azores and Madeira Archipelagos

**DOI:** 10.3897/zookeys.1053.64268

**Published:** 2021-08-02

**Authors:** António Onofre Soares, Hugo Renato Calado, José Carlos Franco, António Franquinho Aguiar, Miguel M. Andrade, Vera Zina, Olga M.C.C. Ameixa, Isabel Borges, Alexandra Magro

**Affiliations:** 1 Centre for Ecology, Evolution and Environmental Changes and Azorean Biodiversity Group, Faculty of Sciences and Technology, University of the Azores, 9500-321, Ponta Delgada, Portugal; 2 Azorean Biodiversity Group, Faculty of Sciences and Technology, University of the Azores, 9501-801, Ponta Delgada, Portugal; 3 Centro de Estudos Florestais (CEF), Instituto Superior de Agronomia, Universidade de Lisboa, 1349-017, Lisboa, Portugal; 4 Laboratório de Qualidade Agrícola, Caminho Municipal dos Caboucos, 61, 9135-372, Camacha, Madeira, Portugal; 5 Rua das Virtudes, Barreiros Golden I, Bloco I, R/C B, 9000-645, Funchal, Madeira, Portugal; 6 Centre for Environmental and Marine Studies and Department of Biology, University of Aveiro, Campus Universitário de Santiago, 3810-193, Aveiro, Portugal; 7 Laboratoire Evolution et Diversité biologique, UMR 5174 CNRS, UPS, IRD, 118 rt de Narbonne Bt 4R1, 31062, Toulouse cedex 9, France; 8 University of Toulouse – ENSFEA, 2 rt de Narbonne, Castanet-Tolosan, France

**Keywords:** Azores, Coccinellidae, Madeira, Palearctic Region, Portugal

## Abstract

A comprehensive annotated checklist of the ladybeetle species of Portugal, including the Azores and Madeira archipelagos, is presented. The Coccinellidae fauna comprises a total of 101 species: 83 from the Mainland, 39 from Madeira, and 32 from the Azores. The listed species are distributed among 2 sub-families and 13 tribes: within the subfamily Microweiseinae, Madeirodulini (1 species), Serangiini (2 species), and within the subfamily Coccinellinae, Azyini (1 species), Chilocorini (4 species), Coccidulini (7 species), Coccinellini (30 species), Epilachnini (4 species), Hyperaspidini (7 species), Noviini (2 species), Platynaspini (1 species), Scymnini (37 species), Stethorini (3 species), and Sticholotidini (2 species). The Portuguese fauna comprises 10 exotic species: 5 present in the Mainland, 7 in Madeira, and 6 in the Azores. *Harmoniaaxyridis* (Pallas, 1773) from Madeira, *Propyleaquatuordecimpunctata* (Linnaeus, 1758) from the Azores, *Delphastuscatalinae* (Horn, 1895) from the Azores and Madeira, Nephus (Geminosipho) reunioni (Fürsch, 1974) and Nephus (Nephus) voeltzkowi Weise, 1910 from Madeira and *Microserangium* sp. from the Mainland, are reported for the first time. Some species are considered doubtful records, as explained in the text. These results were obtained by compiling information on the available literature regarding ladybeetle species on the Portuguese mainland and insular territories, and original data.

## Introduction

The book by Raimundo and Alves (1986) was the last review of the coccinellid (Coleoptera: Coccinellidae) fauna of Portugal. Since then, several studies on Portuguese ladybeetles have been published, including catalogues (e.g. Fürsch 1987; Kovář 2007; Eizaguirre 2015), new individual records (e.g., Serrano and Borges 1987; Raimundo 1992; Soares et al. 2003a, b; Raimundo et al. 2006; Soares et al. 2006), and studies on ladybeetle communities in agricultural ecosystems (e.g. Magro et al. 1994; Carlos et al. 2005; Silva et al. 2006; Silva et al. 2010; Benhadi-Marin et al. 2011; Santos et al. 2012), among others. However, the information is scattered and therefore difficult to analyse.

Ladybeetles comprise about 375 genera (Nedvěd 2020) and nearly 6000 species (Vandenberg 2002), distributed worldwide. They are characterised by a high diversity as regards to their life history, development, distribution, habitat, and food relationships (see Hodek et al. 2012 for review). This family of insects is very charismatic, in particular because most species are predators recognised as useful natural enemies of pests, including aphids (Aphidoidea), scale insects (Coccoidea), whiteflies (Aleyrodoidea) or mites (Acari). Largely used in biological control programmes, ladybeetles render important ecosystem services to agriculture and silviculture (Ameixa et al. 2018). In fact, the first successful case of classical biological control dates back to the late 1800s, when *Novius* (=*Rodolia*) *cardinalis* (Mulsant) was introduced in California from Australia to control the cottony cushion scale, *Iceryapurchasi* Maskell, also of Australian origin (Caltagirone et al. 1989). The introduction of this ladybeetle in Portugal in 1897 was the first case of classical biological control in Europe (Amaro 1994). However, ladybeetles also have other food habits and a few species are herbivores causing heavy crop damages in Asia, America and Africa (Barrigossi et al. 2003; Beyene et al. 2007; Das et al. 2012).

Scientific evidence shows that human activities have huge negative impacts on biodiversity (e.g., Vitousek et al. 1997; Newbold et al. 2015; Jung et al. 2019). The structure and composition of insect communities have been particularly affected by these activities, with ecological domino effects along trophic chains (Dyer et al. 2003). Consequently, action in favour of insect conservation and recovery has been claimed (e.g., Harvey et al. 2020). Ladybeetle communities are no exception. For instance, several studies report long-term variations in the composition of ladybeetle communities following the introduction of *Harmoniaaxyridis* Pallas around the world (Roy et al. 2016). Honӗk et al. (2014, 2017) included agricultural intensification, habitat (particularly urbanization) and climate changes as additional causes for ladybeetle community changes. In this context, and because it is important that each country should have an updated list of its fauna, from which we can detect changes in biodiversity and implement conservation and recovery programmes, we herein address the issue of the coccinellid fauna of Portugal, within the research project AZORESBIOPORTAL–PORBIOTA (ACORES-01-0145-FEDER-000072).

Based on the literature and unpublished data, we present an updated, comprehensive checklist of ladybeetles of Portugal, including the Azores and Madeira Archipelagos.

## Materials and methods

In this work we follow the suprageneric classification of Coccinellidae proposed by Che et al. (2021). This recent revised classification recognises three subfamilies: Microweiseinae, Monocoryninae stat. nov., and Coccinellinae. It should be noted that the tribe Coccidulini*sensu*Seago et al. (2011), which comprises several species for Portugal, was redefined by Che et al. (2021) in which Scymnini and Stethorini were split independently.

Current taxonomic affiliation follows Kovář (2007). Species cited for Portugal after Kovář (2007) or those for which the taxonomic position has changed as a result of more recent studies, are indicated here following more recent publications.

The species are listed in alphabetic order according to their valid tribe and genera. Species of the genera *Nephus* and *Scymnus* are listed under the respective subgenera because they are particularly important for their identification. Synonymy is mentioned but is restricted to the original name.

For each species, we specify the regions where the species were recorded (Mainland, Azores and/or Madeira), under “Distribution” and we provide brief notes about the status of the species in each region, possible taxonomic incongruences, and the current biogeographical (limits as in Löbl and Smetana 2007) distribution under “Comments”. New records are marked with a black spot (^●^), doubtful records with an asterisk (*) and exotic species with a dollar ($). The bibliographic references associated with each species recorded for Portugal are presented in Table 1.

**Table 1.** Checklist (by alphabetic order) and bibliographic references of Coccinellidae species recorded for Portugal, including the Azores and Madeira.

Species	References
*Adaliabipunctata* (Linnaeus, 1758)	12, 27, 29, 30, 32, 39, 42, 47, 50, 52, 53, 55, 59, 60, 64, 67, 68, 74, 75, 77
*Adaliadecempunctata* (Linnaeus, 1758)	3, 4, 5, 9, 12, 14, 26, 30, 32, 39, 42, 47, 50, 52, 53, 55, 56, 57, 59, 60, 64, 65, 67, 68, 72, 73, 74, 75, 77, 82
*Adaliatestudinea* (Wollaston, 1854)	1, 2, 8, 14, 29, 67, 68
*Anatisocellata* (Linnaeus, 1758)	12, 67, 77
*Anisostictanovemdecimpunctata* (Linnaeus, 1758)	13, 12, 39, 67, 77
*Calviadecemguttata* (Linnaeus, 1767)	39, 67, 77
*Calviaquatuordecimguttata* (Linnaeus, 1758)	12, 39, 77
*Calviaquindecimguttata* (Fabricius, 1777)	39
*Ceratomegillanotata* (Laicharting, 1781)	12, 39, 67
*Ceratomegillaundecimnotata* (Schneider, 1792)	12, 39, 55, 67, 77
*Chilocorusbipustulatus* (Linnaeus, 1758)	9, 12, 14, 26, 27, 29, 30, 39, 47, 50, 52, 53, 55, 59, 60, 64, 65, 66, 67, 68, 72, 75
*Chnootribaelaterii* (Rossi, 1794)	31, 39, 67, 77
*Clitostethusarcuatus* (Rossi, 1794)	1, 2, 8, 20, 29, 30, 39, 40, 47, 50, 52, 53, 55, 56, 57, 59, 64, 67, 68, 73, 75
*Coccidularufa* (Herbst, 1783)	18, 39, 77
*Coccidulascutellata* (Herbst, 1783)	12, 39, 77
*Coccinellagenistae* Wollaston, 1854	1, 2, 8, 14, 17, 29, 37, 42, 52, 67, 68
*Coccinellaseptempunctata* Linnaeus, 1758	9, 30, 32, 35, 39, 43, 47, 50, 55, 56, 57, 59, 60, 64, 66, 72, 73, 74, 75, 77
*Coccinellaundecimpunctata* Linnaeus, 1758	3, 4, 5, 9, 30, 32, 35, 39, 45, 55, 59, 67, 77, 82
*Coccinulaquatuordecimpustulata* (Linnaeus, 1758)	1, 2, 39
*Coccinulasinuatomarginata* (Faldermann, 1837)	29, 67
*Coelopterussalinus Mulsant* & Rey, 1852	67
*Cryptolaemusmontrouzieri* Mulsant, 1853	39, 47, 50, 52, 53, 64
*Delphastuscatalinae* (Horn, 1895)	Present study
*Eriopisconnexa* (Germar, 1824)	44, 55, 59
*Exochomusquadripustulatus* (Linnaeus, 1758)	19, 39, 47, 50, 52, 53, 64, 66, 67, 72, 75
*Halyziasedecimguttata* (Linnaeus, 1758)	12, 19, 39, 67, 77
*Harmoniaaxyridis* (Pallas, 1773)	Present study
*Harmoniaquadripunctata* (Pontoppidan, 1763)	29, 30, 39, 40, 42, 47, 50, 64, 67, 68, 77
*Henosepilachnaangusticollis* (Reiche, 1862)	77
*Henosepilachnaargus* (Geoffrey, 1785)	12, 39, 67
*Hippodamiavariegata* (Goeze, 1777)	1, 2, 8, 11, 14, 21, 23, 25, 29, 32, 36, 39, 42, 50, 52, 53, 55, 56, 57, 64, 67, 68, 72, 75, 77
*Hyperaspisconcolor* (Suffrian, 1843)	67, 77
*Hyperaspisduvergeri* Fürsch, 1985	70
*Hyperaspishoffmannseggi* (Gravenhorst, 1807)	39
*Hyperaspisillecebrosa* Mulsant, 1846	16, 67, 77
*Hyperaspispantherina* Fürsch, 1975	58, 61, 68, 69
*Hyperaspisreppensis* (Herbst, 1783)	12, 47, 50, 52, 53, 60, 64, 66
*Hyperaspisstigma* (Olivier, 1808)	67
*Iberorhyzobiusrondensis* (Eizaguirre, 2004)	62, 63, 74, 77, 78, 79
*Madeirodulaatlantica* Szawaryn, Větrovec & Tomaszewska, 2020	85
*Microserangium* sp.	Present study
*Myrrhaoctodecimguttata* (Linnaeus, 1758)	6, 8, 14, 20, 29, 36, 38, 39, 42, 55, 59, 65, 67, 68,75, 77
*Myziaoblongoguttata* (Linnaeus, 1758)	39, 66
Nephus (Bipunctatus) bisignatus (Boheman, 1850)	38, 40, 49, 50, 52, 53, 55, 59, 64, 67, 72, 75, 77
Nephus (Bipunctatus) conjunctus (Wollaston, 1870)	48, 49, 50, 52, 53, 64, 67, 68, 77
Nephus (Bipunctatus) peyerimhoffi (Sicard, 1923)	49, 50, 64, 67, 77
Nephus (Geminosipho) reunioni (Fürsch, 1974)	47, 48, 49, 50, 52, 53, 64, 67, 71, 77
Nephus (Nephus) binotatus (Brisout de Barneville, 1863)	39, 47, 52, 53, 64
Nephus (Nephus) flavopictus (Wollaston, 1854)	1, 2, 8, 14, 15, 17, 21, 22, 25, 30, 26, 27, 29, 32, 35, 40, 41, 42, 46, 55, 57, 59, 67, 68, 83
Nephus (Nephus) quadrimaculatus (Herbst, 1783)	39, 64
Nephus (Nephus) schatzmayri Canepari & Tedeschi, 1977	67, 70
Nephus (Nephus) ulbrichi Fürsch, 1977	49, 52, 53, 64
Nephus (Nephus) voeltzkowi Weise, 1910	84
Nephus (Sidis) depressiusculus (Wollaston, 1867)	46, 68
Nephus (Sidis) hiekei (Fürsch, 1965)	49, 50, 52, 53, 55, 56, 57, 64, 72, 77
Nephus (Sidis) pooti Fürsch, 1999	77
*Noviuscardinalis* (Mulsant, 1850)	30, 32, 35, 39, 42, 44, 45, 46, 47, 49, 50, 52, 53, 54, 55, 60, 64, 68 73, 67, 82
*Noviuscruentatus Mulsant*, 1846	67
*Oenopiaconglobata* (Linnaeus, 1758)	12, 39, 47, 50, 52, 53, 59, 63, 65, 73, 74, 76
*Oenopiadoublieri* (Mulsant, 1846)	12, 39, 50, 52, 53, 64, 67, 72, 77, 81
*Oenopialyncea* (Olivier, 1808)	12, 39, 67
*Parexochomusnigripennis* (Erichson, 1843)	67
*Parexochomusnigromaculatus* (Goeze, 1777)	12, 39, 47, 50, 52, 53, 60, 64, 66, 72, 74, 75
*Pharoscymnusdecemplagiatus* (Wollaston, 1857)	2, 7, 14, 29, 32, 42, 46, 55, 67, 68, 82
*Platynaspisluteorubra* (Goeze, 1777)	12, 39, 47, 50, 52, 53, 64, 72, 75
*Propyleaquatuordecimpunctata* (Linnaeus, 1758)	11, 39, 47, 50, 52, 53, 60, 64, 67, 72, 73, 75
*Psylloboravigintiduopunctata* (Linnaeus, 1758)	12, 39, 47, 50, 52, 53, 67, 77
*Rhyzobiuschrysomeloides* (Herbst, 1792)	10, 12, 36, 39, 47, 50, 52, 53, 55, 56, 57, 64, 65, 66, 67, 68, 72, 73, 74, 75, 82
*Rhyzobiusforestieri* (Mulsant, 1853)	80
*Rhyzobiuslitura* (Fabricius, 1787)	1, 2, 3, 4, 5, 7, 8, 9, 11, 14, 25, 26, 27, 29, 30, 32, 35, 36, 39, 42, 47, 50, 52, 53 55, 56, 57, 64, 65, 67, 68, 72, 73, 75
*Rhyzobiuslophanthae* (Blaisdell, 1892)	36, 39, 42, 44, 46, 47, 50, 51, 52, 53, 55, 56, 59, 64, 65, 66, 67, 68, 72
*Scymniscusfuerschi* (Plaza, 1981)	50, 52, 64
*Scymniscushelgae* (Fürsch, 1965)	38, 39, 40, 55, 59, 72, 74, 75
*Scymniscussemirufus* (Weise, 1885)	39, 47, 48, 64, 72, 77
Scymnus (Mimopullus) epistemoides Wollaston, 1867	10, 14, 29, 30, 40, 66, 67
Scymnus (Mimopullus) limnichoides Wollaston, 1854	1, 2, 8, 14,.30, 40, 51, 67, 68
Scymnus (Mimopullus) marinus (Mulsant, 1850)	1, 2, 8, 12, 14, 30, 39, 40, 47, 50, 51, 52, 53, 64, 65, 68, 74, 75
Scymnus (Neopullus) ater Kugelann, 1794	13, 34, 39, 77
Scymnus (Neopullus) haemorrhoidalis Herbst, 1797	2, 8, 14, 40, 41, 55, 67, 68.
Scymnus (Neopullus) limbatus Stephens, 1832	27, 29, 67, 68
Scymnus (Parapullus) abietis (Paykull, 1798)	2, 11, 39, 56, 57, 68, 77
Scymnus (Pullus) auritus Thunberg, 1795	12, 39, 49, 50, 52, 53, 64, 67, 77
Scymnus (Pullus) subvillosus (Goeze, 1777)	1, 2, 8, 9, 11, 12, 14, 26, 29, 32, 34, 35, 39, 43, 47, 50, 52, 53, 55, 59, 64, 65, 66, 67, 68, 72, 74, 75, 82
Scymnus (Pullus) suturalis Thunberg, 1795	24, 12, 27, 29, 30, 38, 39, 40, 46, 47, 50, 51, 52, 53, 55, 59, 73, 66, 67, 68, 82
Scymnus (Scymnus) apetzi Mulsant, 1846	1, 2, 4, 8, 14, 25, 27, 29, 32, 39, 40, 42, 46, 47, 50, 52, 53, 55, 64, 67, 68, 72, 74, 75
Scymnus (Scymnus) bivulnerus Baudi di Selve, 1894	39, 77
Scymnus (Scymnus) frontalis (Fabricius, 1787)	12, 39, 60, 64, 67, 75, 77
Scymnus (Scymnus) interruptus (Goeze, 1777)	5, 11, 32, 35, 39, 40, 43, 47, 50, 52, 53, 55, 57, 60, 64, 66, 67, 72, 74, 75, 82
Scymnus (Scymnus) laetificus Weise, 1879	77
Scymnus (Scymnus) nubilus Mulsant, 1850	2, 20, 30, 38, 39, 40, 43, 50, 52, 53, 55, 56, 57, 64, 65, 67, 68, 82
Scymnus (Scymnus) rubromaculatus (Goeze, 1777)	27, 29, 30, 32, 40, 55, 67, 68
Scymnus (Scymnus) rufipes (Fabricius, 1798)	12, 39, 47, 54, 52, 53, 60, 64, 72, 67, 77
Scymnus (Scymnus) schmidti Fürsch, 1958	40, 55, 77
Scymnus (Scymnus) suffrianioides Sahlberg, 1913	33, 39, 64, 72
*Sospitavigintiguttata* (Linnaeus, 1758)	12, 39, 77
*Stethoruspusillus* (Herbst, 1797)	9, 19, 20, 26, 32, 35, 39, 40, 47, 50, 52, 53, 55, 59, 60, 64, 72, 73, 74, 75, 82
*Stethorustenerifensis* Fürsch, 1987	42, 51, 67
*Stethoruswollastoni* Kapur, 1948	1, 2, 7, 8, 14, 15, 22, 28, 29, 32, 56, 57, 67, 68
*Subcoccinellavigintiquatuorpunctata* (Linnaeus, 1758)	12, 39, 50, 52, 53, 67, 72
*Tytthaspissedecimpunctata* (Linnaeus, 1761)	12, 39, 52, 53, 67, 73, 75
*Vibidiaduodecimguttata* (Poda von Neuhaus, 1761)	12, 39, 67, 75

1. Wollaston (1854), 2. Wollaston (1857), 3. Drouet (1859), 4. Drouet (1861), 5. Tarnier (1861), 6. Wollaston (1862), 7. Wollaston (1864), 8. Wollaston (1865), 9. Crotch (1867), 10. Wollaston (1867), 11. Hayden (1870), 12. Oliveira (1894), 13. Barros (1896), 14. Fauvel (1897), 15. Cameron (1901), 16. Barros (1913), 17. Winkler (1924-1932), 18. Barros (1926), 19. De la Fuente (1928), 20. De la Fuente (1929), 21. Uyttenboogaart (1930 in Fürsch 1966), 22. Korschefky (1931), 23. Alluaud (1935), 24. Liebmann, (1939), 25. Jansson (1940), 26. Méquignon (1942), 27. Uyttenboogaart (1947), 28. Kapur (1949), 29. Lundblad (1958), 30. Bielawski (1963), 31. Fürsch (1964), 32. Fürsch (1966), 33. Capra and Fürsch (1967), 34. Gourreau (1974), 35. Serrano (1982), 36. Israelson (1984), 37. Mitter (1984), 38. Gillerfors (1986), 39. Raimundo and Alves (1986), 40. Fürsch (1987), 41. Serrano and Borges (1987), 42. Erber and Hinterseher (1988), 43. Borges and Serrano (1989), 44. Borges (1990a), 45. Borges (1990b), 46. Erber (1990), 47. Franco et al. (1992), 48. Magro et al. (1992), 49. Raimundo (1992), 50. Magro et al. (1994), 51. Erber and Aguiar (1996), 52. Magro et al. (1999), 53. Magro and Hemptinne (1999), 54. Soares et al. (1999), 55. Soares et al. (2003a), 56. Soares et al. (2003b), 57. Soares et al. (2003c), 58. Félix et al. (2004), 59. Borges et al. (2005b), 60. Carlos et al. (2005), 61. Félix et al. (2005), 62. Branco et al. (2006), 63. Raimundo et al. (2006), 64. Silva et al. (2006), 65. Soares et al. (2006), 66. Gonçalves et al. (2007), 67. Kovář (2007), 68. Boieiro et al. (2008), 69. Félix et al. (2008), 70. Canepari (2009), 71. Borges et al. (2010), 72. Santos et al. (2010), 73. Silva et al. (2010), 74. Benhadi-Marin et al. (2011), 75. Santos et al. (2012), 76. Tavares et al. (2014), 77. Eizaguirre (2015), 78. Tavares et al. (2015a), 79. Tavares et al. (2015b), 80. Borges et al. (2017), 81. Borges et al. (2018), 82. Calado (2018), 83. Romanowski et al. (2019), 84. Magro et al. (2020b), 85. Szawaryn et al. (2020).

## Results

### Subfamily MICROWEISEINAE

#### Tribe Madeirodulini

***Madeirodulaatlantica* Szawaryn, Větrovec and Tomaszewska 2020 (following Szawaryn et al. 2020)**

**Distribution.** Madeira.

**Comments.** This is a recently described new tribe, genus and species, endemic to Madeira.

#### Tribe SERANGIINI

**^$^*Delphastuscatalinae* (Horn, 1895)**

= *Cryptognathacatalinae*Horn 1895

**Distribution.** Madeira^●^ and Azores^●^.

**Comments.** A native species of Nearctic and Neotropical regions, currently established in the Palearctic region following introductions in biological control programs. Its presence in the Azores probably originated from deliberate releases for biological control of whiteflies. A large population was first recorded by Isabel Borges, from kales in a vegetable garden (S. Miguel Island 37°48'02"N, 25°36'42"W), August 2018, where both adults and larvae were abundant. In Madeira, Délia Cravo collected in October 2006 from *Musaacuminata* Colla (Funchal 32°39'26"N, 16°55'56"W) and José Jesus collected in September 2020 on *Citrusreticulata* Blanco, (Santana 32°48'27"N, 16°53'13"W). First records for Portugal.

**^$^*Microserangium* sp.**

= *Microserangium* Miyatake, 1961

**Distribution.** Mainland**^●^**.

**Comments.** Oriental origin. First observations by Vera Zina, in 2012, Algarve. A few individuals were collected in August 2012, and May, July and November 2013 from the canopy of citrus (Carocha, Boliqueime 37°08'55.9"N, 8°08'11.6"W; Estibeira, Boliqueime 37°07'27"N, 8°07'16"W; Benafim 37°14'17"N, 8°06'36"W). First record for Portugal.

### Subfamily COCCINELLINAE

#### Tribe AZYINI

**^$^*Cryptolaemusmontrouzieri* Mulsant, 1853**

**Distribution.** Mainland.

**Comments.** Currently established in Palearctic, Afrotropical, Nearctic and Neotropical regions. This exotic species of Australian origin, used around the world for biological control since the 19^th^ century, was introduced from France in the early 20^th^ century for the biological control of the citrus mealybug, *Planococcuscitri* (Risso) (Franco et al. 1994), but the first record in Europe was in Italy, 1908 (Roy and Migeon 2010).

#### Tribe Chilocorini

***Chilocorusbipustulatus* (Linnaeus, 1758)**

= *Coccinellabipustulata* Linnaeus, 1758

**Distribution.** Mainland, Madeira and Azores.

**Comments.** Palearctic, Afrotropical, and Nearctic distribution.

***Exochomusquadripustulatus* (Linnaeus, 1758)**

= *Coccinellaquadripustulata* Linnaeus, 1758

**Distribution.** Mainland.

**Comments.** Palearctic and Nearctic distribution.

***Parexochomusnigripennis* (Erichson, 1843)**

= *Chilocorusnigripennis* Erichson, 1843

**Distribution.** Mainland.

**Comments.** Palearctic and Afrotropical distribution.

***Parexochomusnigromaculatus* (Goeze, 1777)**

= *Coccinellanigromaculata* Goeze, 1777

**Distribution.** Mainland

**Comments.** Palearctic distribution.

#### Tribe Coccidulini

***Coccidularufa* (Herbst, 1783)**

= *Dermestesrufus* Herbst, 1783

**Distribution.** Mainland.

**Comments.** Palearctic distribution.

***Coccidulascutellata* (Herbst, 1783)**

= *Chrysomelascutellata* Herbst, 1783

**Distribution.** Mainland.

**Comments.** Palearctic distribution.

***Iberorhyzobiusrondensis* (Eizaguirre, 2004)**

= *Coccidularondensis* Eizaguirre, 2004

**Distribution.** Mainland.

**Comments.** This is an endemic species of the Iberian Peninsula, associated with maritime pine forests, and is a specialist predator of the maritime pine bast scale, *Matsucoccusfeytaudi* Ducasse (Tavares et al. 2014; Tavares et al. 2015a; Tavares et al. 2015b). Adults and mostly larvae were shown to be attracted by the sex pheromone of their prey (Branco et al. 2006).

***Rhyzobiuschrysomeloides* (Herbst, 1792)**

= *Strongyluschrysomeloides* Herbst, 1792

**Distribution.** Mainland, Madeira and Azores.

**Comments.** Palearctic distribution.

**^$^*Rhyzobiusforestieri* (Mulsant, 1853)**

= *Platyomusforestieri* Mulsant, 1853

**Distribution.** Azores.

**Comments.** Palaearctic, Nearctic and Australian distribution. This exotic species of Australian origin was introduced in Europe in the 1980´s for the biological control of scale insects (Coccoidea), and became established in different countries, including Italy, France, Greece and Albania (Roy and Migeon 2010; Soares et al. 2018). The first record in Europe was in Italy in 1982 (Roy and Migeon 2010). It was recently recorded in the Azores (Borges et al. 2017).

***Rhyzobiuslitura* (Fabricius, 1787)**

= *Nitidulalitura* Fabricius, 1787

**Distribution.** Mainland, Madeira and Azores.

**Comments.** Palearctic distribution.

**^$^*Rhyzobiuslophanthae* (Blaisdell, 1892)**

= *Scymnuslophanthae* Blaisdell, 1892

**Distribution.** Mainland, Madeira and Azores.

**Comments.** Palaearctic, Afrotropical, Nearctic, Neotropical, and Australian distribution. This species, native to Queensland, Australia (Tomaszewska 2010) was first introduced in Europe (Italy), in 1908, for the biological control of armoured scale insects (Coccoidea: Diaspididae) and imported to Portugal in the 1930’s and 1980’s (Roy and Migeon 2010).

#### Tribe Coccinellini

***Adaliabipunctata* (Linnaeus, 1758)**

= *Coccinellabipunctata* Linnaeus, 1758

**Distribution.** Mainland, Madeira and Azores.

**Comments.** Worldwide distributed (Palaearctic, Afrotropical, Australian, Nearctic and Neotropical regions).

***Adaliadecempunctata* (Linnaeus, 1758)**

= *Coccinelladecempunctata* Linnaeus, 1758

**Distribution.** Mainland, Madeira and Azores.

**Comments.** Palearctic distribution.

***Adaliatestudinea*** (*Wollaston, 1854*)

= *Coccinellatestudinea* Wollaston, 1854

**Distribution.** Madeira.

**Comments.** Macaronesian endemic species.

***Anatisocellata* (Linnaeus, 1758)**

=*Coccinellaocellata* Linnaeus, 1758

**Distribution.** Mainland.

**Comments.** Palearctic distribution.

***Anisostictanovemdecimpunctata* (Linnaeus, 1758)**

= *Coccinellanovemdecimpunctata* Linnaeus, 1758

**Distribution.** Mainland.

**Comments.** Palearctic distribution.

***Calviadecemguttata* (Linnaeus, 1767)**

= *Coccinelladecemguttata* Linnaeus, 1767

**Distribution.** Mainland.

**Comments.** Palearctic distribution.

***Calviaquatuordecimguttata* (Linnaeus, 1758)**

= *Coccinellaquatuordecimguttata* Linnaeus, 1758

**Distribution.** Mainland.

**Comments.** Palearctic, Nearctic and Oriental distribution.

***Calviaquindecimguttata* (Fabricius, 1777)**

= *Coccinellaquindecimguttata* Fabricius, 1777

**Distribution.** Mainland.

**Comments.** Palearctic distribution.

***Ceratomegillanotata* (Laicharting, 1781)**

= *Coccinellanotata* Laicharting, 1781

**Distribution.** Mainland.

**Comments.** Palearctic distribution.

***Ceratomegillaundecimnotata* (Schneider, 1792)**

= *Coccinellaundecimnotata* Schneider, 1792

**Distribution.** Mainland and Azores.

**Comments.** Palearctic distribution.

***Coccinellagenistae* Wollaston, 1854**

**Distribution.** Madeira.

**Comments.** Macaronesian endemic species.

***Coccinellaseptempunctata* Linnaeus, 1758**

**Distribution.** Mainland, Madeira and Azores*.

**Comments.** Palearctic, Afrotropical, Nearctic and Oriental distribution. Relatively important species in cereal crops in the Azores, especially in the first half of the 20^th^ century, having eventually disappeared when these crops became scarce (Soares et al. 2008; Soares et al. 2017). The taxonomic status of *C.algerica* has been under discussion because of its morphological similarities to the geographically widespread *Coccinellaseptempunctata* L. Although Lecompte et al. (2016) revealed a high genetic structuring pattern, with an Algerian rear-edge population highly differentiated, consistent with their morphological distinctiveness, a recent study by Romanowski et al. (2019) demonstrated that individuals from Canarian populations, usually classified as *C.algerica*, can hybridise with individuals from European populations of *C.septempunctata* giving rise to fertile F1 descendants. These authors therefore propose to synonymise *C.algerica* with *C.septempunctata* but, taking into account the morphological peculiarities of the North African and the Canarian populations, they consider that this species is a subspecies: *Coccinellaseptempunctataalgerica* Kovář, 1977.

***Coccinellaundecimpunctata* Linnaeus, 1758**

**Distribution.** Mainland and Azores.

**Comments.** Palaeartic, Australian and Nearctic distribution. In the Azores, it is a threatened species due to anthropogenic pressures on the coastal areas (Soares et al. 2017).

***Coccinulaquatuordecimpustulata* (Linnaeus, 1758)**

= *Coccinellaquatuordecimpustulata* Linnaeus, 1758

**Distribution.** Mainland and Madeira*.

**Comments.** Palearctic and Afrotropical distribution. Although previously recorded in the Madeira archipelago, there are doubts regarding its present occurrence. It might have been introduced but did not establish (Franquinho Aguiar, personal communication).

***Coccinulasinuatomarginata* (Faldermann, 1837)**

= *Coccinellasinuatomarginata* Faldermann, 1837

**Distribution.** Mainland and Madeira*.

**Comments.** Palearctic distribution. Although previously recorded for the Madeira archipelago, there are doubts as to its present occurrence. It might have been introduced but did not establish (Franquinho Aguiar, personal communication).

**^$^*Eriopisconnexa* (Germar, 1824)**

= *Coccinellaconnexa* Germar, 1824

**Distribution.** Azores*.

**Comments.** Of Neotropical origin where it is very common. Although previously reported for the Azores, it did not become established (*fide* A. O. Soares, after intensive surveys).

***Halyziasedecimguttata* (Linnaeus, 1758)**

= *Coccinellasedecimguttata* Linnaeus, 1758

**Distribution.** Mainland.

**Comments.** Palearctic distribution.

**^$^*Harmoniaaxyridis* (Pallas, 1773)**

= *Coccinellaaxyridis* Pallas, 1773

**Distribution.** Madeira^●^.

**Comments.** Worldwide distribution. Several specimens (adults, larvae and pupae) collected by Miguel M. Andrade, in September 2019, from *Enterolobium* sp. (Funchal 32°38'39"N, 16°55'31"W), Graça Freitas and Franquinho Aguiar, collected in September and October 2020, from *Annonacherimola* Mill. (Funchal 32°39'47"N, 16°53'41"W). *Harmoniaaxyridis* is the most invasive insect of the world (Roy et al. 2016). However, despite deliberate attempts to introduce the species in the Azores, she has not became established. The apparent failure can be explained by a combination of resource availability and inter-specific competition (Soares et al. 2017; Soares et al. 2018) and climate conditions (Alaniz et al. 2021). The fate of this introduction in Madeira, whose conditions are like those of the Azores, will be important to follow. First record for Portugal.

***Harmoniaquadripunctata* (Pontoppidan, 1763)**

= *Coccinellaquadripunctata* Pontoppidan, 1763

**Distribution.** Mainland, Madeira.

**Comments.** Palearctic and Nearctic distribution.

***Hippodamiavariegata* (Goeze, 1777)**

= *Coccinellavariegata* Goeze, 1777

**Distribution.** Mainland, Madeira and Azores.

**Comments.** Palearctic, Afrotropical, Nearctic and Oriental distribution.

***Myrrhaoctodecimguttata* (Linnaeus, 1758)**

= *Coccinellaoctodecimguttata* Linnaeus, 1758

**Distribution.** Mainland, Madeira and Azores.

**Comments.** Palearctic distribution.

***Myziaoblongoguttata* (Linnaeus, 1758)**

= *Coccinellaoblongoguttata* Linnaeus, 1758

**Distribution.** Mainland.

**Comments.** Palearctic distribution.

***Oenopiaconglobata* (Linnaeus, 1758)**

= *Coccinellaconglobata* Linnaeus, 1758

**Distribution.** Mainland.

**Comments.** Palearctic distribution.

***Oenopiadoublieri* (Mulsant, 1846)**

= *Harmoniadoublieri* Mulsant, 1846

**Distribution.** Mainland and Azores.

**Comments.** Palearctic distribution.

***Oenopialyncea* (Olivier, 1808)**

= *Coccinellalyncea* Olivier, 1808

**Distribution.** Mainland.

**Comments.** Palearctic distribution.

***Propyleaquatuordecimpunctata* (Linnaeus, 1758)**

= *Coccinellaquatuordecimpunctata* Linnaeus, 1758

**Distribution.** Mainland and Azores^●^.

**Comments.** Palearctic and Nearctic distribution. First record for the Azores. Several adults were collected in July 2019 by António O. Soares and Isabel Borges, in a vegetable garden, of the parish of Castelo Branco (GPS coordinates: 38°31'23.2"N, 28°41'21.0"W), Faial Island.

***Psylloboravigintiduopunctata* (Linnaeus, 1758)**

= *Coccinellavigintiduopunctata* Linnaeus, 1758

**Distribution.** Mainland.

**Comments.** Palearctic distribution.

***Sospitavigintiguttata* (Linnaeus, 1758)**

= *Coccinellavigintiguttata* Linnaeus, 1758

**Distribution.** Mainland.

**Comments.** Palearctic distribution.

***Tytthaspissedecimpunctata* (Linnaeus, 1761)**

= *Coccinellasedecimpunctata* Linnaeus, 1761

**Distribution.** Mainland.

**Comments.** Palearctic distribution.

***Vibidiaduodecimguttata* (Poda von Neuhaus, 1761)**

= *Coccinelladuodecimguttata* Poda von Neuhaus, 1761

**Distribution.** Mainland.

**Comments.** Palearctic and Oriental distribution.

#### Tribe Epilachnini

***Chnootribaelaterii* (Rossi, 1794)**

= *Coccinellaelaterii* Rossi, 1794

**Distribution.** Mainland.

**Comments.** Palearctic and Afrotropical distribution. Reported by Fürsch (1964) as the subspecies portugalensis. Based on molecular and morphological data, it was suggested to transfer this species to the genus *Chnootriba* (Szawaryn et al. 2015; Tomaszewska and Szawaryn 2016).

***Henosepilachnaangusticollis* (Reiche, 1862)**

= *Epilachnaangusticollis* Reiche, 1862

**Distribution.** Mainland.

**Comments.** Distributed in the Mediterranean region.

***Henosepilachnaargus* (Geoffrey, 1785)**

= *Coccinellaargus* Geoffrey, 1785

**Distribution.** Mainland.

**Comments.** Palearctic and Afrotropical distribution.

***Subcoccinellavigintiquatuorpunctata* (Linnaeus, 1758)**

= *Coccinellavigintiquatuorpunctata* Linnaeus, 1758

**Distribution.** Mainland.

**Comments.** Palearctic and Nearctic distribution.

#### Tribe Hyperaspidini

***Hyperaspisconcolor* (Suffrian, 1843)**

= *Coccinellaconcolor* Suffrian, 1843

**Distribution.** Mainland.

**Comments.** Palearctic distribution.

***Hyperaspisduvergeri* Fürsch, 1985**

**Distribution.** Mainland.

**Comments.** Palearctic distribution.

***Hyperaspishoffmannseggi* (Gravenhorst, 1807)**

= *Coccinellahoffmannseggi* Gravenhorst, 1807

**Distribution.** Mainland.

**Comments.** Palearctic distribution.

***Hyperaspisillecebrosa* Mulsant, 1846**

**Distribution.** Mainland.

**Comments.** Palearctic distribution. Eizaguirre (2015) refers to the existence of two subspecies for the Iberian Peninsula; *illecebrosa* Mulsant, 1846 and *castiliana* Eizaguirre ssp. nov.; only the first one is mentioned for Portugal.

**^$^*Hyperaspispantherina* Fürsch, 1975, following Félix et al. (2004), Félix et al. (2005), Félix et al. (2008)**

**Distribution.** Madeira.

**Comments.** Established in Palearctic/Madeira, originally from Afrotropical region. The first record for Europe was in 2002, in Madeira archipelago (Roy and Migeon 2010). In Madeira, it was introduced as a biological control agent against *Insignortheziainsignis* (Browne 1887).

***Hyperaspisreppensis* (Herbst, 1783)**

= *Coccinellareppensis* Herbst, 1783

**Distribution.** Mainland*.

**Comments.** Palearctic distribution. According to Eizaguirre (2015), this species does not exist in the Iberian Peninsula and has been mistaken for the endemic species, *H.illecebrosa*.

***Hyperaspisstigma* (Olivier, 1808)**

= *Coccinellastigma* Olivier, 1808

**Distribution.** Mainland.

**Comments.** Palearctic distribution.

#### Tribe Noviini

**^$^*Noviuscardinalis* (Mulsant, 1850), following Pang et al. (2020)**

= *Vedaliacardinalis* Mulsant, 1850

**Distribution.** Mainland, Madeira and Azores.

**Comments.** Established in Palaeartic, Afrotropical, Nearctic and Neotropical, Oriental. Native to Australian region. This exotic species was introduced in California and South Africa in the 1890´s for the control of the cottony cushion scale, *Iceryapurchasi* Maskell (Roy and Migeon 2010). The first introduction in Europe was made in Portugal, 1897 (Amaro 1994; Roy and Migeon 2010). *Rodoliacardinalis* is the widely known name and it was included in the genus *Novius* by Pang et al. (2020).

***Noviuscruentatus* Mulsant, 1846**

**Distribution.** Mainland.

**Comments.** Palearctic distribution.

#### Tribe Platynaspidini

***Platynaspisluteorubra* (Goeze, 1777)**

= *Coccinellaluteorubra* Goeze, 1777

**Distribution.** Mainland.

**Comments.** Palearctic distribution.

#### Tribe SCYMNINI

***Clitostethusarcuatus* (Rossi, 1794)**

=*Coccinellaarcuata* Rossi, 1794

**Distribution.** Mainland, Madeira and Azores.

**Comments.** Palearctic, Afrotropical, Nearctic distribution.

**Nephus (Bipunctatus) bisignatus (Boheman, 1850)**

= *Scymnusbisignatus* Boheman, 1850

**Distribution.** Mainland and Azores.

**Comments.** Palearctic distribution.

**Nephus (Bipunctatus) conjunctus (Wollaston, 1870)**

= *Scymnusconjunctus* Wollaston, 1870

**Distribution.** Mainland and Madeira.

**Comments.** Palearctic and Afrotropical distribution. This species was first reported as N. (bipunctatus) includens (Kirsch 1871) based on adults emerged from larvae collected in 1988 from citrus fruits infested with mealybugs, in the South of Portugal (Algarve) (Raimundo 1992). However, Eizaguirre (2015) indicates that N. (bipunctatus) includens is a junior synonym of *N.conjuntus*. Taking this into account and knowing that specimens previously collected in 1984 in the Algarve and identified as *N.quadrimaculatus* Herbst were in fact shown to be *N.includens* (Raimundo and Alves 1986; Magro et al. 1992; Raimundo 1992), we have to consider that the first report of this species dates from 1984. The distribution is apparently restricted to the Algarve (Magro et al. 1992).

**Nephus (Bipunctatus) peyerimhoffi (Sicard, 1923)**

= *Scymnuspeyerimhoffi* Sicard, 1923

**Distribution.** Mainland.

**Comments.** Palearctic and Afrotropical distribution.

**^$^Nephus (Geminosipho) reunioni (Fürsch, 1974a)**

= *Scymnusreunioni* Fürsch, 1974a

**Distribution.** Mainland, Azores and Madeira^●^.

**Comments.** Palearctic and Afrotropical distribution. The first record in Europe was in France in 1983 (Roy and Migeon 2010). This species of Afrotropical origin was imported from France (laboratory rearing in Antibes) in the early 1980´s and released in Oeiras, in 1984 (Magro et al. 1992). Its presence in Portugal was detected for the first time in 1990, in citrus orchards in the Setúbal region (Franco et al. 1992). In his Catalogue of the African species of the genus *Nephus*, Fürsch (2007) mentions that the distribution of *N.reunioni* is apparently restricted to Reunion Island and Mauritius, and that the references to its presence in South Africa and other Mediterranean countries, such as Portugal and Israel, are due to misidentifications by the author himself, which most likely correspond to *N.derroni*Fürsch 1974b, a species described from São Tomé Island and that is common in South Africa. However, Magro et al. (2020a) analysed specimens from Portugal and showed that they corresponded to the original description of *N.reunioni* made by Fürsch in Chazeau et al. (1974). Raimundo (1992), who first described *N.reunioni* for Portugal, also illustrated the external morphology and genitalia corresponding to the original description by Fürsch in Chazeau et al. (1974). In both cases, the observations showed that the specimens from the Portuguese population are distinct from *N.derroni*. In Madeira, collected by Aguiar and Jesus, in October 2008, from *Dombeyawallichii* (Lindl.) Baill. (Funchal 32°38'49"N, 16°56'16"W), Celestina Brazão in February 2003, from *Dombeyawallichii* (Lindl.) Baill. (Funchal 32°39'49"N, 16°55'44"W), Aguiar and Jesus, in April 2005, from *Oleaeuropaea* L. (Machico 32°38'49"N, 16°56'16"W), Délia Cravo, in September 2006, from *Jacarandamimosifolia* D. Don (Funchal 32°39'05"N, 16°54'18"W), J.D. Sardinha, in January 2010, from *Perseaamericana* Mill. (Funchal 32°39'30"N, 16°54'34"W), Graça Freitas, in August 2011, from *Perseaamericana* Mill. (Funchal 32°39'00"N, 16°53'27"W), Graça Freitas, in July 2013, from *Perseaamericana* Mill. (Calheta 32°42'23"N, 17°08'39"W), Paula Rocha, in January 2015, from *Annonacherimola* Mill. (Funchal 32°39'47"N, 16°50'40"W), Florasanto, in June 2015, from *Pinuspinaster* Aiton (São Vicente 32°47'46"N, 17°01'55"W), Natália Nunes, in January 2016, from *Laurusnovocanariensis* Rivas Mart., Lousã, Fern. Prieto, E. Díaz, J.C. Costa & C. Aguiar (Ponta do Sol 32°40'57"N, 17°06'01"W), Celestina Brazão, in August 2017, from *Neriumoleander* L. (Funchal 32°38'11"N, 16°56'02"W), Fátima Rocha, in November 2019, from *Psidiumguajava* L. (Funchal 32°39'34"N, 16°52'33"W), Graça Freitas, in September 2020, from *Annonacherimola* Mill. (Funchal 32°39'47"N, 16°53'40"W), and Alexandra Magro and Miguel Sequeira, in September 2018, from herbaceous plants (Anjos 32°69'11"N, 17°11'96"W and Ribeira de Natal, Caniçal 32°73'57"N, 16°74'62"W). New record for Madeira.

**Nephus (Nephus) binotatus (Brisout de Barneville, 1863)**

= *Scymnusbinotatus* Brisout de Barneville, 1863

**Distribution.** Mainland.

**Comments.** Palearctic distribution.

**Nephus (Nephus) flavopictus (Wollaston, 1854)**

= *Scymnusflavopictus* Wollaston, 1854

**Distribution.** Madeira and Azores.

**Comments.** Macaronesian endemic species.

**Nephus (Nephus) quadrimaculatus (Herbst, 1783)**

= *Sphaeridiumquadrimaculatum* Herbst, 1783

**Distribution.** Mainland.

**Comments.** Palearctic distribution.

**Nephus (Nephus) schatzmayri Canepari & Tedeschi, 1977**

**Distribution.** Mainland.

**Comments.** Palearctic distribution.

**Nephus (Nephus) ulbrichi Fürsch, 1977**

**Distribution.** Mainland.

**Comments.** Palearctic distribution.

**^$^Nephus (Nephus) voeltzkowi Weise, 1910, following Magro et al. (2020b)**

**Distribution.** Azores and Madeira^●^.

**Comments.** Afrotropical origin. Very recently, two parthenogenetic populations of this species were found in the Azores and Mascarene archipelagos, becoming the first reported case of asexuality in the Coccinellidae (Magro et al. 2020b). Observations by António Onofre Soares, in September of 1997, Madeira Island (Anjos; approximately at 32°41'15"N, 17°06'54"W; Faial approximately 32°47'24"N, 16°51'02"W; Caniçal approximately 32°44'49"N, 16°44'26"W), and Alexandra Magro and Miguel Sequeira in September 2018, from herbaceous plants (Anjos 32°69'11"N, 17°11'96"W; Ribeira de Natal, Caniçal 32°73'57"N, 16°74'62"W; Ribeira Brava 32°66'98"N, 17°06'09"W; Fajã dos Padres 32°65'45"N, 17°02'13"W). New record for Madeira.

**Nephus (Sidis) depressiusculus (Wollaston, 1867)**

= *Scymnusdepressiusculus* Wollaston, 1867

**Distribution.** Madeira.

**Comments.** Palearctic and Afrotropical distribution.

**Nephus (Sidis) hiekei (Fürsch, 1965)**

= *Scymnushiekei* Fürsch, 1965

**Distribution.** Mainland.

**Comments.** Palearctic distribution.

**Nephus (Sidis) pooti Fürsch, 1999**

**Distribution.** Mainland.

**Comments.** Palearctic distribution.

***Scymniscusfuerschi* (Plaza, 1981)**

= *Nephusfuerschi* Plaza, 1981

**Distribution.** Mainland.

**Comments.** Palearctic distribution.

***Scymniscushelgae* (Fürsch, 1965)**

= *Scymnushelgae* Fürsch, 1965

**Distribution.** Mainland and Azores.

**Comments.** Palearctic distribution.

***Scymniscussemirufus* (Weise, 1885)**

=*Scymnussemirufus* Weise, 1885

**Distribution.** Mainland.

**Comments.** Palearctic distribution.

**Scymnus (Mimopullus) epistemoides Wollaston, 1867**

**Distribution.** Madeira.

**Comments.** Palearctic distribution.

**Scymnus (Mimopullus) limnichoides Wollaston, 1854**

**Distribution.** Madeira.

**Comments.** Palearctic distribution.

**Scymnus (Mimopullus) marinus (Mulsant, 1850)**

= *Rhyzobiusmarinus* Mulsant, 1850

**Distribution.** Mainland and Madeira.

**Comments.** Palearctic distribution.

**Scymnus (Neopullus) ater Kugelann, 1794**

**Distribution.** Mainland.

**Comments.** Palearctic distribution.

**Scymnus (Neopullus) haemorrhoidalis Herbst, 1797**

**Distribution.** Madeira and Azores.

**Comments.** Palearctic distribution.

**Scymnus (Neopullus) limbatus Stephens, 1832**

**Distribution.** Madeira.

**Comments.** Palearctic distribution.

**Scymnus (Parapullus) abietis (Paykull, 1798)**

= *Coccinellaabietis* Paykull, 1798

**Distribution.** Mainland and Madeira.

**Comments.** Palearctic distribution.

**Scymnus (Pullus) auritus Thunberg, 1795**

**Distribution.** Mainland.

**Comments.** Palearctic distribution.

**Scymnus (Pullus) subvillosus (Goeze, 1777)**

= *Coccinellasubvillosa* Goeze, 1777

**Distribution.** Mainland, Madeira and Azores.

**Comments.** Palearctic and Afrotropical distribution.

**Scymnus (Pullus) suturalis Thunberg, 1795**

**Distribution.** Mainland, Madeira and Azores.

**Comments.** Palearctic and Nearctic distribution.

**Scymnus (Scymnus) apetzi Mulsant, 1846**

**Distribution.** Mainland.

**Comments.** Palearctic distribution.

**Scymnus (Scymnus) bivulnerus Baudi di Selve, 1894**

**Distribution.** Mainland.

**Comments.** Palearctic distribution.

**Scymnus (Scymnus) frontalis (Fabricius, 1787)**

= *Coccinellafrontalis* Fabricius, 1787

**Distribution.** Mainland.

**Comments.** Palearctic distribution.

**Scymnus (Scymnus) interruptus (Goeze, 1777)**

= *Coccinellainterrupta* Goeze, 1777

**Distribution.** Mainland, Madeira and Azores.

**Comments.** Palearctic distribution.

**Scymnus (Scymnus) laetificus Weise, 1879**

**Distribution.** Mainland.

**Comments.** Palearctic distribution. However, it only occurs in the Western Mediterranean.

**Scymnus (Scymnus) nubilus Mulsant, 1850**

**Distribution.** Mainland, Madeira and Azores.

**Comments.** Palearctic distribution. On Portugal’s mainland, this species was wrongly identified as *Scymnuslevaillanti* Mulsant.

**Scymnus (Scymnus) rubromaculatus (Goeze, 1777)**

= *Coccinellarubromaculata* Goeze, 1777

**Distribution.** Mainland, Madeira and Azores.

**Comments.** Palearctic and Afrotropical distribution.

**Scymnus (Scymnus) rufipes (Fabricius, 1798)**

= *Coccinellarufipes* Fabricius, 1798

**Distribution.** Mainland.

**Comments.** Palearctic distribution.

**Scymnus (Scymnus) schmidti Fürsch, 1958**

**Distribution.** Mainland and Azores.

**Comments.** Palearctic distribution.

**Scymnus (Scymnus) suffrianioides Sahlberg, 1913**

**Distribution.** Mainland.

**Comments.** Palearctic distribution.

#### Tribe Stethorini

***Stethoruspusillus* (Herbst, 1797)**

= *Scymnuspusillus* Herbst, 1797

**Distribution.** Mainland and Azores.

**Comments.** Palearctic distribution.

***Stethorustenerifensis* Fürsch, 1987**

**Distribution.** Madeira.

**Comments.** Macaronesian endemic species.

***Stethoruswollastoni* Kapur, 1948**

**Distribution.** Madeira.

**Comments.** Macaronesian endemic species.

#### Tribe Sticholotidini

***Coelopterussalinus* Mulsant & Rey, 1852**

**Distribution.** Mainland.

**Comments.** Palearctic and Afrotropical distribution.

***Pharoscymnusdecemplagiatus* (Wollaston, 1857)**

= *Scymnusdecemplagiatus* Wollaston, 1857

**Distribution.** Madeira and Azores.

**Comments.** Palearctic distribution.

## Acknowledgments

In Memoriam of Armando Américo Cardoso Raimundo (2.V.1942–9.Xi.2019): A farewell to colleague and friend.

Thanks are due to Claudio Canepari for helping in the identification of *Microserangium* sp. and Jaroslav Větrovec of *Harmoniaaxyridis* Pallas from Madeira.

This study was financed by FEDER in 85% and by Azorean Public funds by 15% through Operational Program Azores 2020, under the following projects AZORESBIOPORTAL –PORBIOTA (ACORES-01-0145-FEDER-000072), and under the project ECO2 – TUTA (ACORES-01-0145-FEDER-000081). The Forest Research Centre is funded by Fundação para a Ciência e a Tecnologia I.P. (FCT), Portugal (UIDB/00239/2020). Thanks are also due to FCT/MCES for financial support to CESAM (UIDB/50017/2020+UIDP/50017/2020) through national funds, and the co-funding by the FEDER, within the PT2020 Partnership Agreement and Compete 2020. OMCCA is funded by national funds (OE), through FCT, in the scope of the framework contract foreseen in the numbers 4, 5 and 6 of the article 23, of the Decree-Law 57/2016, of August 29, changed by Law 57/2017, of July 19. AM was supported by the “Laboratoires d’Excellence” LabEx TULIP (ANR-10-LABX-41). The Open Access of this manuscript was supported by the project FCT-UIDB/00329/2020-2024.
